# Production and validation of durable, high quality standardized malaria microscopy slides for teaching, testing and quality assurance during an era of declining diagnostic proficiency

**DOI:** 10.1186/1475-2875-5-92

**Published:** 2006-10-25

**Authors:** Jason D Maguire, Edith R Lederman, Mazie J Barcus, Wendy A Prudhomme O'Meara, Robert G Jordon, Socheat Duong, Sinuon Muth, Priyanto Sismadi, Michael J Bangs, W Roy Prescott, J Kevin Baird, Chansuda Wongsrichanalai

**Affiliations:** 1 U.S. Naval Medical Research Unit No.2 (NAMRU-2), Jakarta, Indonesia; 2 Hydas Incorporated, Hershey, Pennsylvania, USA; 3 National Institutes of Health, Bethesda, Maryland, USA; 4 National Center for Parasitology, Entomology and Malaria Control (CNM), Phnom Penh, Cambodia; 5 National Institute of Health Research and Development, Ministry of Health, Jakarta, Indonesia; 6 Naval Medical Center Portsmouth, 620 John Paul Jones Circle, Portsmouth, Virginia 23708-2197, USA

## Abstract

**Background:**

Sets of Giemsa-stained, blood smear slides with systematically verified composite diagnoses would contribute substantially to development of externally validated quality assurance systems for the microscopic diagnosis of malaria.

**Methods:**

whole blood from *Plasmodium*-positive donors in Cambodia and Indonesia and individuals with no history of risk for malaria was collected. Using standard operating procedures, technicians prepared Giemsa-stained thick and thin smears from each donor. One slide from each of the first 35 donations was distributed to each of 28 individuals acknowledged by reputation as having expertise in the microscopic diagnosis of malaria. These reference readers recorded presence or absence of *Plasmodium *species and parasite density. A composite diagnosis for each donation was determined based on microscopic findings and species-specific small subunit ribosomal RNA (ssrRNA) DNA polymerase chain reaction (PCR) amplification.

**Results:**

More than 12, 000 slides were generated from 124 donations. Reference readers correctly identified presence of parasites on 85% of slides with densities <100 parasites/μl, which improved to 100% for densities >350 parasites/μl. Percentages of agreement with composite diagnoses were highest for *Plasmodium falciparum *(99%), followed by *Plasmodium vivax *(86%).

**Conclusion:**

Herein, a standardized method for producing large numbers of consistently high quality, durable Giemsa-stained blood smears and validating composite diagnoses for the purpose of creating a malaria slide repository in support of initiatives to improve training and competency assessment amidst a background of variability in diagnosis is described.

## Background

Despite advances in antigen detection and gene sequence amplification technology, microscopic examination of Giemsa-stained blood film is still the gold standard for malaria diagnosis. Early malariologists, including Ronald Ross, emphasized the importance of externally validating results and training microscopists [1]. Decades later, during the height of the global malaria eradication campaign of the 1950s and 1960s, experts recognized that "as malaria begins to disappear from each country, case finding and parasite identification become essential in locating residual foci of transmission, the halting of which achieves eradication. In searching this out, the microscopist plays a key role" [2]. This becomes increasingly relevant as malaria reemerges in areas where it was previously controlled, like Java [3] or the highlands of western Kenya [4]. As multi-drug resistant falciparum malaria continues to emerge and new regimens are developed for differential treatment of *Plasmodium falciparum *and other species, accurate species detection becomes critical [5] and the importance of competency in microscopic diagnosis assumes substantial new weight.

The quality of microscopy is highly variable and methods for validating proficiency are not standardized. Currently described methods for slide preparation [2,6,7] yield well known variation in smear readability as well as parasite quantification. Reporting of results varies between institutions and depends on the skills of the microscopists, quality of equipment and setting, i.e. research versus clinical case management [8]. The current lack of standardized, externally validated approaches to proficiency testing contributes to the risk of diagnostic error [9].

Most recommendations for quality assurance procedures in microscopy date back to the days of global eradication efforts. In countries where malaria eradication programme failed, skills at the healthcare periphery, where the vast majority of patients seek medical care, and within the reference laboratories themselves, have not been adequately sustained. The emergence of the new malaria control paradigm, focusing on treatment rather than prevention [10], demands resolve to ensure minimum standards of competency at all levels of microscopic diagnostic services.

The lack of acknowledged standards of competency and a method for ascertaining individual competency imposes one of the primary obstacles to reliable diagnosis. Production of a collection of Giemsa-stained blood films, each having meticulously vetted composite diagnoses, as described herein, is the first step in addressing this problem by. This collection offers a true "gold standard" for assessments of reader competency as well as sensitivity and specificity for other diagnostic modalities. Such rigor can serve as the basis for externally validated competency at laboratories responsible for compiling and distributing blood films for international, national, local and certification programmes.

## Methods

### Subjects, screening, enrollment and donation

Donors were selected to support the primary objective of creating a slide repository. In Cambodia, adults attending health centers with acute fever served as the sources of donors. In Indonesia, people participating in Institutional Review Board (IRB)-approved research protocols or in malariometric surveys volunteered to participate. All donors positive for plasmodia were treated immediately following donation of blood according to prescribed practice in each setting or according to the IRB-approved research protocols in which they were participating. Unambiguously malaria negative donors were restricted to natives of a malaria-free country without travel to a malaria endemic area for the past two years or donating blood within five days of any possible exposure to risk of infection, e.g., a person arriving in Indonesia from the United States. Project protocols received ethical review approvals by the National Ethics Committee for Health Research (NEC) of the Cambodian Ministry of Health and the National Institute of Health Research and Development of the Indonesian Ministry of Health. After donors provided informed consent, technicians collected 3 cc of whole blood, transferred it to an EDTA-containing glass test tube and mixed it by gentle inversion.

### Slide preparation

Smears were prepared on pre-cleaned and uncoated glass slides with frosted ends (Goldseal^® ^Rite-on superthin microslides, 75 × 25 mm). Technicians washed all new slides by soaking them in a tub of soapy water for at least 2 hours. After rinsing with tap water, each slide was individually dried with cotton gauze or a lint-free towel and placed on a hot plate (50–60°C) until completely dry. Slides were stored in plastic boxes containing silica gel. Just prior to blood collection, the donor's sequential identity number was written in pencil on the frosted end of each slide. Blood was always placed on the slides within 6 hours of collection. Technicians never processed slides from more than one donor at any given time and never more than three donors per day.

Technicians used a precision microtiter pipette with sterile tip to place 6 μl toward the labeled end of the slide and 2 μL in the central part of the slide, and immediately prepared thick and thin films, respectively. With the aid of a paper template visible through the slide, technicians placed each drop at the same place on every slide. They prepared thick and thin smears immediately prior to any drying of the blood on the slides. One technician transferred the blood drop to the slide and another prepared the smear in an "assembly line" fashion. To make the thick smear, the technician used the corner of a clean slide to gently swirl the drop of blood to form an even circle of 12 mm diameter outlined in a paper template. If bubbles were present, the technician stirred the thick smear again until no bubbles remained. Technicians prepared thin smears using a clean spreader slide according to standard methods [7] to create a feathered edge. Blood smears dried overnight at room temperature in a horizontal position before being further processed for staining. This was done to ensure complete drying, which in the authors' experience with anticoagulated blood, reduces the otherwise high frequency of thick smear separation from the slide during the staining process.

### Slide staining

Only slides from the same donor were stained in the same batch. Slides from malaria negative donors were stained using equipment specifically reserved for that purpose. A fresh batch of 5% (1:20 dilution in bottled drinking water, pH 7.2) Merck Giemsa Solution (Azur-Eosin-Methylene blue Solution for Microscopy, Merck KgaA, 64271 Darmstadt, Germany) was prepared just prior to staining each set of slides. After overnight drying, technicians fixed thin smears by dipping in a container of absolute methanol, avoiding contact between methanol and the thick film. After a few minutes drying, slides were arranged in a staining tray for batch Giemsa staining (maximum 50 slides per batch). After 30 minutes at room temperature, technicians removed the slide trays from the Giemsa solution and gently swirled them in a container of tap water before placement in a slotted plastic or wooden block for drying at room temperature.

### Cover-slipping

Technicians placed cover slips over the thick and thin smear to increase shelf life. Once the Giemsa-stained slides dried, they were stored in clean, plastic slide boxes. Within two weeks, technicians prepared them for batch cover-slipping using Poly-Mount^® ^(Polysciences, Inc., Warrington, Pennsylvania) mounting medium, 1–2 ml transferred to a clean glass test tube (single use for each session). Using an Eppendorf pipettor, technicians transferred 100 μL of Poly-Mount^® ^to each slide as a thin line running lengthwise from the frosted end to the feathered edge of the thin film, without allowing the pipette tip to touch the stained blood film. To minimize air bubbles under the cover slips (Goldseal^® ^Cover Glass, 24 × 50 mm), technicians gently lowered them to the surface of the slides from roughly a 45° angle slowly into the horizontal position. Technicians then applied gentle pressure to the cover slips to spread mounting medium evenly and wiped excess mounting medium from the edges of the slides. Slides dried in ambient air overnight before labeling and storage in slide boxes. Each slide was labeled with a unique encrypted 8 digit identifier, which was printed in numeric and barcode format (Loftware 2000, Loftware Inc., Maine) so that it could not be linked to a specific donor by readers.

### Polymerase chain reaction (PCR)

At the time of venous blood sampling, blood blots were collected onto Whatman No. 1 filter paper (Whatman International, Maidstone, Kent, UK) for drying and storage. Any whole blood remaining after slide preparation was stored at -70°C. DNA was extracted from dried blood blot samples using Chelex^® ^100 5% resin (Bio-Rad^® ^Laboratories Inc. Hercules, CA) in distilled water. Samples were incubated for 20 minutes on a 56°C heat block followed by eight minutes on a 100°C heat block. Diagnostic PCR amplification of the gene for small-subunit ribosomal RNA (ssrRNA DNA) was conducted using the methods of Kimura *et al*. [11] to detect all four species of plasmodia.

### Reference reader composite microscopy

After obtaining a provisional microscopic diagnosis by staff microscopists, 30 slides from each of 35 donors, without providing the diagnoses, were referred for reference reader evaluation and ultimately, determination of a composite diagnosis for each donor blood sample. After soliciting referrals from experts in the malaria research community, 43 individuals were invited to participate in the study as reference readers. All twenty-eight (65%) who agreed to participate were selected as reference readers. They represented 13 countries on five continents. Confidentiality of the reference readers was guaranteed and all understood they were among a pool of other reference readers unknown to them. Reference readers were aware that positive slides were collected in Cambodia and Indonesia but were unaware of what proportion of the slides they reviewed was positive. Slides sent to each reference reader contained one slide from each of the 35 donations.

Reference readers were provided guidelines for slide reading methodology and asked them to record presence of malaria parasites, species, stages, and parasite counts using a standard electronic form that automatically calculated the parasite density (per μl) based on the total number of asexual stage parasites and WBC counted in the high power thick film fields and a standard multiplier of 8,000 WBC/μl. Although the method for enumerating parasites was not mandated, the WBC method [12] was recommended unless the number of parasites on thick smear exceeded the number of WBCs by a factor of 5 or more. In such cases, they were asked to use the red blood cell count method, whereby the number of parasites per microliter was equal to percent parasitized erythrocytes on the thin smear multiplied by 4,500,000. To avoid the difficulties associated with differential counting of ring and early trophozoite forms of different species, since other species indicators (RBC cell size, stippling, etc.) visible on the thin film are not evident on the thick smear, reference readers were asked to report combined asexual stage parasite densities when more than one species of plasmodia was identified. No deadline was imposed upon the reference readers, nor dictated time or field examination per reading.

Reported results of the reference readers were compiled to complete two distinct tasks: 1) identifying unqualified reference readers with consistently incompatible results relative to other readers and 2) deriving a composite diagnosis for as many of the donations as possible based on analysis of the combined results of the reference readers and PCR analysis of the particular donation. The composite diagnosis was to be accepted as the true diagnosis. The objective was simply to assemble a collection of blood films for which composite diagnoses could be accepted as accurate. In the event of a high degree of discordance among readers for a particular donation, those slides were excluded as inherently ambiguous and not suitable for use in the slide sets. The process of both qualifying reference readers and establishing a composite diagnosis among them is described below.

Three tiers of diagnostic proficiency were established and classified by assigning demerit points based on types of errors made (Table 1). First (primary), whether the reported result was positive or negative for plasmodia was considered. A false positive was considered a more serious error (10 demerits) than a false negative (5 demerits). Second (secondary), diagnosis by species was considered, which can be either simply wrong (e.g., calling *P. falciparum *parasitaemia infection by *P. vivax*) or partially correct by degree when dealing with mixed infections with one species having an extremely low count. Finally (tertiary), the parasite counts were considered, for which individual accuracy was ascertained by accepting the median count among qualified readers as the best estimate of true count. A primary diagnosis was accepted as the composite diagnosis with >80% accordance among readers. Among positive smears, the composite secondary diagnosis required >80% accordance on single species diagnoses. When readers identified a mixed infection, even if numerically in the minority, and that secondary diagnosis was affirmed by PCR analysis, their view was accepted as the composite diagnosis. When PCR analysis detected the presence of a species not identified by any of the reference readers, the composite diagnosis was assigned based on microscopic observation alone, and the PCR result was attributed to a second infection below the limit of microscopic detection.

**Table 1:** Diagnostic classification of reference reader results used to establish accepted composite diagnosis and identify unqualified readers

**Level of Diagnosis**	**Reported Diagnosis**	**Variable**	**Composite Diagnosis**	**Misdiagnosis Demerit Points**
Primary	Plasmodia positive or negative	Dichotomous	Agreement >80%	10: false positive 5: false negative
Secondary	Species	Discontinuous (Pf, Pv, Pm, Po)*	Agreement >80% for mono-infection Agreement >50% for mixed infection**	3: infection misclassified
Tertiary	Parasite Count	Continuous	Median count	1: parasite density outside 99% CI

* Pf = *Plasmodium falciparum*, Pv = *P. vivax*, Pm = *P. malariae*, Po = *P. ovale*, includes any combination of these species

** or less than 50% with PCR confirmation

## Results

### Enrollment and slide production

One hundred and twenty-four adults donated blood and 12,362 slides were accessioned into the repository. After accessioning, 114 (0.92%) slides were discarded on the basis of poor quality. Based on availability and slide set requirements, the first 35 of the 124 donations were selected for the rigorous process of external validation by composite diagnosis among reference readers.

### Reference reader performance

**Figure 1:**
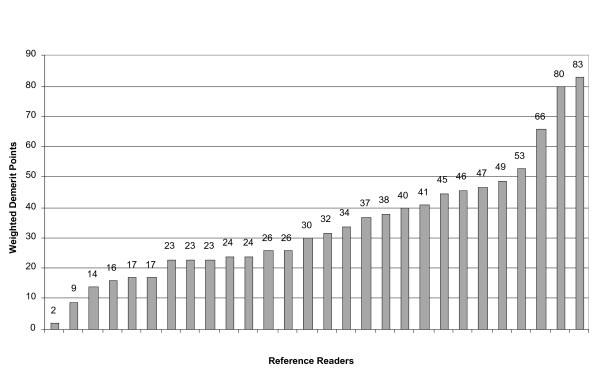
Weighted misdiagnosis demerit points for 28 reference readers after independently reading Giemsa smears from 35 donors.

Among the 28 reference readers, individual cumulative diagnostic demerit points ranged from two to 83 (mean 33, SD 17). The findings of 4 readers with demerit points greater than one standard deviation above the mean from further analysis were excluded (Figure 1). Table 2 summarizes the results for each of the 35 donations subjected to external validation by the remaining 24 readers. In comparison to the assigned composite diagnoses for the 24 donations for which diagnoses were established, reference readers correctly identified presence of parasites (primary level of diagnosis) 85% of the time when parasite densities were <100 parasites per μl. Percentage of correct primary diagnosis improved at higher densities, 99% for densities between 100 and 350/μl and 100% for densities >350/μl. μl. Correct primary diagnosis improved at higher densities, 99% for densities between 100 and 350/μl and 100% for densities >350/μl. Reference readers correctly identified 96% of true negative slides. They correctly identified *P. falciparum*, *P. vivax *and *P. malariae *mono-infections (secondary level of diagnosis) 99%, 86% and 50% of the time, respectively. Mixed infections were identified in only 47% of the time, usually a consequence of low density parasitaemia of the second species not reported. This observation was most notable for donation 12 (Table 2), for which only 13% of reference readers (and PCR) identified *P. vivax *at low density. Among the eight *P. vivax *composite diagnoses, five were misdiagnosed as *P. ovale *by between one and four reference readers.

**Table 2:** Giemsa smear results among 24 remaining reference readers for 35 donations subjected to external validation

**Donor**	**Composite Diagnosis**	**Reader Agreement %**	**PCR Result**	**Reference Reader Results**	**Median Parasite Density (per ul)**
12	Pf/Pv	13	PfPv	3Pf/Pv, 21Pf	31,222
8	Pm	50	Pm	12Pm, 2Pm/Pv, 2Pv, 2Pf, 5neg, 1 no sp	95
16	Pm	58	Pm	14Pm, 10Pf/Pm	3,183
6	Pf/Pv	63	PfPv	15Pf/Pv, 6Pf, 3Pf/Po	15,084
11	Pf/Pv	67	PfPv	16 Pf/Pv, 6Pv, 1Pf/Po, 1Pf/Pm/Pv	129,437
50	Pv	83	Pv	20Pv, 3Po, 1Po/Pv	14,895
2	Pv	83	Pv	20Pv, 4Pf/Pv	15,000
15	Pv	88	Pv	21Pv, 2Pm, 1neg	64
17	Pv	88	Pv	21 Pv, 2 Po, 1Po/Pv	1,335
49	Pv	88	Pv	21 Pv, 2 Po, 1Pm/Po/Pv	328
4	NEG	92	neg	22neg, 2 Pf	n/a
52	NEG	92	neg	22neg, 1 Pf, 1Pv	n/a
10	Pf	96	Pf	23Pf, 1Pf/Pv	1,620
20	Pf	96	Pf	23Pf, 1 no species	202
21	Pf	96	Pf	23Pf, 1Pf/Pm	165,500
22	NEG	96	neg	23neg,1Pf	n/a
1	Pf	100	Pf	24Pf	15,453
3	Pf	100	Pf	24Pf	3,542
7	Pf	100	Pf	24Pf	3,669
9	Pf	100	Pf	24Pf	340
13	Pf	100	Pf	24Pf	1,048
18	Pf	100	Pf	24Pf	301,996
19	Pf	100	Pf	24Pf	1,404
23	NEG	100	neg	24neg	n/a
44	NEG	100	neg	24neg	n/a
46	Not used*	50	Pf	12Pf, 12 neg	9
58	Not used*	58	Pv	14Pv, 4Po, 2Pm, 1Pm/Po/Pv, 1Pf/Po, 1neg, 1no sp	63
14	Not used*	63	Pv	15Pv, 4Po, 3Pf/Pv, 1Pm/Pv, 1Pm/Po/Pv	5,191
25	Not used*	67	Pf	16Pv, 2Po, 1Pf, 5neg	48
59	Not used*	71	Pv	17Pv, 1Po, 2Pf, 2neg, 1Pf/Po/Pv, 1Pf/Pm/Po	135
60	Not used*	75	Pf	18Pf, 1Po, 3neg, 2no sp	90
54	Not used*	79	Pf	19Pf, 2Pv, 1neg, 2no species	78
55	Pf* not used	83	Pf	20Pf, 1Pv, 1Po, 1Pf/Pm, 1 no species	129
5	Pf* not used	100	Pf	24Pf	2,625
24	Pf* not used	100	Pf	24Pf	10,885

Pf = *Plasmodium falciparum*, Pv = *P. vivax*, Pm = *P. malariae*

* Donation not included in the U.S. National Institutes of Health Malaria Research and Reference Reagent Resource Center (MR4) malaria slide repository either because composite diagnosis could not be determined or because slides not required for sets

### Slide quality

Table 3 summarizes comments on individual slide quality by the reference readers. Negative comments were infrequent. Microscopists reported under-lysis of thick smear red blood cells most frequently (2.85%) followed by presence of artifact or debris (1.65%) or stain deposits (1.4%).

**Table 3:** Prevalence of specific reference reader comments regarding the quality of individual slides read, n = 911

**Comments**	**Number**	**Percent**
Thick smear red blood cells not lysed or under-lysed	26	2.85%
Artifact or debris present	15	1.65%
Stain deposit present	13	1.43%
Understained slide	12	1.32%
Fungal, bacterial or yeast elements present	6	0.66%
Poor thick smear (unqualified)	4	0.44%
Faint or poor stippling	4	0.44%
Poor mount or cover slip broken	3	0.33%
Overstained slide	2	0.22%
Poor stain (unqualified)	2	0.22%
Poor slide (unqualified)	2	0.22%
Slide not readable	1	0.11%
Dirty prep	1	0.11%

### Slide product

Based on the 24 reference reader results, a composite diagnosis was derived, including parasite density, for 28 of the 35 evaluated donations (Table 2). Diagnoses included five negatives, 13 *P. falciparum *(129 – 302,000/μl), five *P. vivax *(64 – 15,000/μl), three mixed *P. falciparum/P. vivax *(15,000 – 129,000/μl) and two *P. malariae *(95 – 3,200/μl). Composite diagnoses were not determined for the seven remaining donations because the reference reader results were equivocal (donations14, 25, 46, 54, 58, 59 and 60). Three of the donations, (donations 5, 24 and 55) were not used for the slide sets because the densities and species they contained were not required. Donation 12 represents the sole deviation from composite diagnosis selection criteria outlined in Table 1. Its inclusion was necessary to meet goals for numbers of slides representing mixed infections in the sets. Despite the fact that only 13% of the reference readers identified the low density *P. vivax *co-infection with *P. falciparum*, a composite diagnosis of mixed *P. falciparum/P. vivax *infection was assigned because: 1) the accuracies of the three reference readers who identified the *P. vivax *were exemplary for all other slides (total weighted misdiagnosis demerit points <25); 2) it is likely that due diligence in identifying the second species on the part of the other 21 microscopists was obviated by presence of *P. falciparum *at such a high density (31,000 parasites/μl) and 3) the presence of *P. vivax *DNA was confirmed by PCR.

From the slides with composite diagnoses, 24 sets of 50 slides each were compiled and provided to the U.S. National Institutes of Health Malaria Research and Reference Reagent Resource Center (MR4, Manassas, VA, USA) with answer keys containing the median parasite densities and 99% CI as a guide for acceptable quantification. Sets contained slides with the following distribution: 25% negative, 19% higher density *P. falciparum *(≥2,500/μl), 18% lower density *P. falciparum *(≤2,000/μl), 10% higher density *P. vivax *(≥5,000/μl), 9% lower density *P. vivax *(≤300/μl), and 19% mixed *P. falciparum/P. vivax *and *P. malariae *slides. These slide sets are now available for distribution to MR4 registered users for a limited use period. Copies of the standard operating procedures (SOPs) used for their creation are also available from MR4.

## Discussion

Using the currently available standard of competency for microscopic diagnosis of malaria, namely reputation, a team of reference readers was assembled and by critically evaluating their performance relative to one another and PCR-based evidence, their level of competency was affirmed. The product of this exercise, the collection of standardized Giemsa-stained blood films described, is as nearly unambiguous as possible with respect to accuracy of diagnoses. This collection, or others similarly prepared, would provide a validated, objective benchmark for evaluating the competency of microscopists in research, clinical and public health settings or the validity of other diagnostic methods.

It is intuitively obvious that the difficulty of any test is dependent upon the ease of the questions being asked, an observation that holds true for practical assessment of proficiency in the microscopic diagnosis of malaria. Even among the panel of experts assembled for this project, rates of agreement with the composite diagnosis were closely tied with the difficulty of the slide under evaluation, presuming that slides with lower density parasitaemia, less commonly encountered species (*P. malariae*) and mixed infections are more difficult. Ultimately, if diagnostic proficiency is to be objectively compared to raise international standards of performance, a slide bank such as the one described here would serve as the only viable means of such a fair comparison, systematically using the same blood with the exact same degree of difficulty.

The findings described here highlight and confirm many of the previously published difficulties facing diagnosis of malaria today. Milne et al. [13] found that single species infections are misdiagnosed about 25% of the time and that incorrect diagnosis of mixed infections is even more frequent, 71%. In terms of parasite density detection threshold, they also reported a ten-fold lower sensitivity at these labs compared to the expected sensitivity of presumed experienced microscopists (50/μl) [13], a finding also observed elsewhere [14,15]. Even among the panel of experts assembled for this project, percentages of agreement with the composite diagnosis were closely tied with the difficulty of the slide under evaluation, presuming that slides with lower density parasitaemia, less commonly encountered species (*P. malariae*) and mixed infections are more difficult. A mean 15% failed to detect parasites when densities were less than 100/μl and only 47% (range 13–67%) accurately diagnosed mixed infections.

The standard operating procedures for this project were derived in an attempt to resolve some of the inherent imprecision associated with the variability in methodology for slide preparation and parasite quantification including the use of precise blood volumes and a thick film template to ensure consistency in blood film thickness [16-18]. Although finger prick specimens are most desirable because they yield blood from capillary rich areas where the density of developed trophozoites or schizonts is greater [19], blood was obtained by venipuncture and collection into anticoagulant-coated tubes because of the large number of slides to be created from each donor. However, through strict logistical and technical arrangements, parasite and blood cell morphologies were preserved with minimal loss due to thick smear detachment. By batching slides from a single donor and reserving designated containers for negative-only donors, contamination between *Plasmodium*-positive and negative blood smears was precluded [20,21]. Permanently mounting slides protected the surface of the smears against damage from multiple uses, and no reference readers reported interference of the cover-slips with microscopic viewing.

Some methods for quantifying parasitaemia may be cumbersome [7]. Using the thin smear red blood cell count method as the benchmark, Greenwood and Armstrong [22] reported more accurate estimation of the parasite density based on the number of parasites per high power field and the assumed volume of blood present when compared to the WBC method [12]. They attributed their finding to low variability in the blood volume used to prepare the thick blood film and high variability of WBC count in the African population studied. A recent comparison of the WBC, RBC and ocular grid methods identified no significant discrepancies among readers using these methods and no significant difference in parasite densities when comparing the WBC and RBC methods [23]. In this study, the WBC method was recommended [12]. Although it may be less accurate for estimating parasite densities, its relative ease in teaching and testing settings is a plus. In the context of the planned use for such slides, accuracy in parasite density is less important than consistency in methodology for interpretation, therefore, precision. As long as end users of these slides employ the WBC method, comparisons of their estimated parasite densities with the composite diagnosis should prove a fair indicator of individual competency.

## Conclusion

No standards currently exist for determining what constitutes competent microscopy, let alone an expert microscopist. The experience described herein with 28 reputed experts, specifically the variability in results observed, highlights this point. Such standards must be established. Production of durable, validated high quality standardized malaria microscopy slides is the first and essential step toward that end. Using the methodologies described herein, institutions from around the world could establish repositories of uniform slide sets to meet the growing demand for verifiable training, testing and quality assurance materials. Ultimately, if diagnostic proficiency is to be objectively compared to raise international standards of performance, a slide bank such as the one described here serves as the only viable means of such a fair comparison, systematically using the same blood with the exact same degree of difficulty.

## Authors' contributions

JDM coordinated project planning and implementation in Indonesia and Cambodia and protocol development at both locations, supervised execution and data management and wrote the manuscript. ERL was the principal investigator in Indonesia, responsible for project execution. MJB coordinated efforts of the expert readers, and was directly involved in data entry and management. WAP conducted statistical analysis. RGJ contributed to project planning and compilation of the microscopist panel. SD was involved in execution of the project in Cambodia.

SM was involved in execution of the project in Cambodia. PS was involved in execution of the project in Indonesia. MJB participated in project planning oversight in Indonesia. WRP participated in project planning and oversight of microscopist team and data flow. JKB provided expert consultation in all phases of project. CW was the principal investigator in Cambodia, responsible for project execution. All authors read and approved the final manuscript.

## Disclaimer

The assertions herein are the views of the authors and do not reflect official policy of the U.S. Department of the Navy or the U.S. Department of Defense.

## References

[B1] Ross R (1903). An improved method for the microscopical diagnosis of intermittent fever. Lancet.

[B2] Wilcox A (1960). Manual for the Microscopical Diagnosis of Malaria in Man.

[B3] Barcus MJ, Laihad F, Sururi M, Sismadi P, Marwoto H, Bangs MJ, Baird JK (2002). Epidemic malaria in the Menoreh hills of Central Java. Am J Trop Med Hyg.

[B4] Shanks GD, Biomndo K, Hay SI, Snow RW (2000). Changing patterns of clinical malaria since 1965 among a tea estate population located in the Kenyan highlands. Trans R Soc Trop Med Hyg.

[B5] Barnish G, Bates I, Iboro J (2004). Newer drug combinations for malaria may be impractical unless diagnostic accuracy can be improved. BMJ.

[B6] Navy Environmental Health Center (1998). Navy Medical Department Pocket Guide to Malaria Prevention and Control.

[B7] WHO (1991). Basic Malaria Microscopy, Part I & II.

[B8] Durrheim DN, Becker PJ, Billinghurst K, Brink A (1997). Diagnostic disagreement – the lessons learnt from malaria diagnosis in Mpumalanga. South Afr Med J.

[B9] Ohrt C, Purnomo Sutamihardja MA, Tang D, Kain K (2002). Impact of microscopy error on estimates of protective efficacy in malaria-prevention trials. J Infect Dis.

[B10] Baird JK (2000). Resurgent malaria at the millennium: control strategies in crisis. Drugs.

[B11] Kimura M, Kaneko O, Liu Q, Zhou M, Kawamoto F, Wataya Y, Otani S, Yamaguchi Y, Tanabe K (1997). Identification of the 4 species of human malaria parasites by nested PCR that targets variant sequences in the small subunit rRNA gene. Parasit Int.

[B12] Warhurst DC, Williams JE (1996). Laboratory diagnosis of malaria. J Clin Pathol.

[B13] Milne LM, Kyi MS, Chiodini PL, Warhurst (1994). Accuracy of routine laboratory diagnosis of malaria in the United Kingdom. J Clin Pathol.

[B14] Lema OE, Carter JY, Nagelkerke N, Wangai MW, Kitenge P, Gikunda SM, Arube PA, Munafu CG, Materu SF, Adhiambo CA, Mukunza HK (1999). Comparison of five methods of malaria detection in the outpatient setting. Am J Trop Med Hyg.

[B15] McKenzie FE, Sirichaisinthop J, Miller RS, Gasser RA, Wongsrichanalai C (2003). Dependence of malaria detection and species diagnosis by microscopy on parasite density. Am J Trop Med Hyg.

[B16] Hanscheid T (1999). Diagnosis of malaria: a review of alternatives to conventional microscopy. Clin Lab Haematol.

[B17] Krauss W (1931). The role of the physician and the value of the thick film in the control of malaria. South Med J.

[B18] Sinton JA, Banerjea AC (1925). Notes on the thick film method of examination of malaria parasites. Ind J Med Res.

[B19] Gilles H, Gilles HM, Warrell DA (1993). Diagnostic methods in malaria. Essential Malariology.

[B20] Moody A (2002). Rapid diagnostic tests for malaria parasites. Clin Microbiol Rev.

[B21] Aubouy A, Carme B (2004). *Plasmodium *DNA contamination between blood smears during Giemsa staining and microscopic examination. J Inf Dis.

[B22] Greenwood BM, Armstrong JR (1991). Comparison of two simple methods for determining malaria parasite density. Trans R Soc Trop Med Hyg.

[B23] Prudhomme O'Meara W, Remich S, Ogutu B, Lucas M, Mtalib R, Obare P, Oloo F, Onoka C, Osoga J, Ohrt C, McKenzie EE Systematic comparison of two methods to measure parasite density from malaria blood smears. Parasitol Res.

